# Resting-State EEG Signal for Major Depressive Disorder Detection: A Systematic Validation on a Large and Diverse Dataset

**DOI:** 10.3390/bios11120499

**Published:** 2021-12-06

**Authors:** Chien-Te Wu, Hao-Chuan Huang, Shiuan Huang, I-Ming Chen, Shih-Cheng Liao, Chih-Ken Chen, Chemin Lin, Shwu-Hua Lee, Mu-Hong Chen, Chia-Fen Tsai, Chang-Hsin Weng, Li-Wei Ko, Tzyy-Ping Jung, Yi-Hung Liu

**Affiliations:** 1 International Research Center for Neurointelligence (WPI-IRCN), The University of Tokyo Institutes for Advanced Study (UTIAS), The University of Tokyo, Tokyo 113-0033, Japan; wu.chiente@mail.u-tokyo.ac.jp; 2 Hipposcreen Neurotech Corp. (HNC), Taipei 114, Taiwan; Alex_Huang@hipposcreen-nc.com (H.-C.H.); alan_huang@hipposcreen-nc.com (S.H.); Daniel_Weng@hipposcreen-nc.com (C.-H.W.); 3 Division of Psychosomatic Medicine, Department of Psychiatry, National Taiwan University Hospital, Taipei 100229, Taiwan; iming@ntuh.gov.tw (I.-M.C.); scliao@ntu.edu.tw (S.-C.L.); 4 Institute of Health Policy and Management, National Taiwan University, Taipei 10617, Taiwan; 5 Department of Psychiatry & Community Medicine Research Center, Chang Gung Memorial Hospital, Keelung 204, Taiwan; kenchen@cgmh.org.tw (C.-K.C.); 8902008@cgmh.org.tw (C.L.); 6 College of Medicine, Chang Gung University, Taoyuan 33302, Taiwan; shlee@cgmh.org.tw; 7 Department of Psychiatry, Chang Gung Memorial Hospital, Taoyuan 33305, Taiwan; 8 Department of Psychiatry, Taipei Veterans General Hospital, Taipei 11217, Taiwan; mhchen12@vghtpe.gov.tw (M.-H.C.); cftsai@vghtpe.gov.tw (C.-F.T.); 9 Faculty of Medicine, National Yang Ming Chiao Tung University, Taipei 11217, Taiwan; 10 Department of Bio Science & Tech., National Yang Ming Chiao Tung University, Hsinchu 30010, Taiwan; lwko@nctu.edu.tw; 11 Institute for Neural Computation, University of California, San Diego, CA 92093, USA; 12 Department of Mechanical Engineering, National Taiwan University of Science and Technology, Taipei 106335, Taiwan

**Keywords:** electroencephalographic (EEG) signal, healthcare, major depressive disorder (MDD), machine learning, support vector machine, feature selection

## Abstract

Major depressive disorder (MDD) is a global healthcare issue and one of the leading causes of disability. Machine learning combined with non-invasive electroencephalography (EEG) has recently been shown to have the potential to diagnose MDD. However, most of these studies analyzed small samples of participants recruited from a single source, raising serious concerns about the generalizability of these results in clinical practice. Thus, it has become critical to re-evaluate the efficacy of various common EEG features for MDD detection across large and diverse datasets. To address this issue, we collected resting-state EEG data from 400 participants across four medical centers and tested classification performance of four common EEG features: band power (BP), coherence, Higuchi’s fractal dimension, and Katz’s fractal dimension. Then, a sequential backward selection (SBS) method was used to determine the optimal subset. To overcome the large data variability due to an increased data size and multi-site EEG recordings, we introduced the conformal kernel (CK) transformation to further improve the MDD as compared with the healthy control (HC) classification performance of support vector machine (SVM). The results show that (1) coherence features account for 98% of the optimal feature subset; (2) the CK-SVM outperforms other classifiers such as K-nearest neighbors (K-NN), linear discriminant analysis (LDA), and SVM; (3) the combination of the optimal feature subset and CK-SVM achieves a high five-fold cross-validation accuracy of 91.07% on the training set (140 MDD and 140 HC) and 84.16% on the independent test set (60 MDD and 60 HC). The current results suggest that the coherence-based connectivity is a more reliable feature for achieving high and generalizable MDD detection performance in real-life clinical practice.

## 1. Introduction

Major depressive disorder (MDD) is a prevalent mood disorder characterized by persistent sadness, psychomotor retardation, and loss of interest in daily activities [1]. MDD is the leading cause of disability [2], with significant implications for the social functioning and work productivity of individuals [3,4]. Over 700,000 people kill themselves every year as a result of MDD, a disease that affects 320 million people worldwide [5]. In particular, the prevalence of MDD in the United States has increased substantially from 7% prior to the COVID-19 pandemic to 27% during the first year of the pandemic (April 2020–March 2021) [6]. Although treatments for MDD are promising, accurate diagnosis may be difficult due to its heterogeneous etiologies and various psychopathological manifestations [7]. Currently, the diagnosis of MDD is still based on clinical interviews and self-reports. There is an urgent need to develop complementary diagnostic tools that differentiate MDD from healthy persons based on neurophysiological changes.

### 1.1. Related Works

Many studies have tried to apply machine learning (ML) methods to classify resting-state (e.g., [8,9,10,11,12,13,14,15,16,17,18,19,20,21,22,23]) versus task-related (e.g., [21,24,25]) electroencephalography (EEG) signals between MDD and healthy control (HC) groups. A few studies have also proposed multi-model systems that fuse EEG signals and other physiological data (e.g., [26]).

Various features of EEG signals have been investigated. For example, in a study by Bachmann et al. [9], a classification accuracy of 92% on single-electrode EEGs of 26 participants was reported based on the use of a combination of spectral features and complexity features such as Higuchi’s fractal dimension (HFD) and detrended fluctuation analysis (DFA). Hosseinifard et al. [23] reported that EEG alpha and beta power features achieved 70% and 73% accuracies, respectively, and that the use of complexity features could achieve greater that 80% accuracy. A study reported 99.6% accuracy [18] by using a combination of statistical, spectral, function connectivity, and complexity features on a public dataset containing 19-channel EEGs from 64 participants.

The reported MDD-HC classification accuracy based on EEG and ML varies greatly across studies (~60–99%). Such variations result from the differences in terms of chosen algorithms (features, classifiers, etc.), and also the differences in terms of apparatus setting, EEG collection procedure, and participant demographics. Even though some of previous studies have reported high classification accuracy, the true generalization performance of previous methods is still unknown, because the EEG datasets used in most of the previous studies were quite small (ours included [20,21]).

### 1.2. Problem Description

The sample sizes in these ML-based studies [9,10,11,12,13,14,15,16,17,18,19,20,21,22,23,24] were all less than 100 (24~90), except for two studies by Cai et al. [12,15] in which the subject numbers were 267 and 213, respectively (however, they only measured a very low-density EEGs from three electrodes at FP1, FP2, and Fz). Previous studies, due to limited data size, have only performed cross validation procedures on limited EEG data to estimate the generalization performance of their proposed algorithms/models, without using an independent test set of sufficiently large size. As a result of this limitation, the cross validation in previous studies often suffered from overfitting due to information and data leakage (more details are explained in Section 2). Furthermore, MDD is a highly heterogeneous disorder [27]. Due to the heterogeneity, it is difficult for a dataset of limited size to faithfully represent the real-life variations in EEG signals. All these factors including the use of small EEG dataset, the overfitting due to the data leakage in the cross validation, and the lack of an independent EEG test set, could lead to an overly optimistic or even biased estimate of performance. Therefore, to develop an EEG-based MDD-HC classification model with high generalizability, increasing the size and diversity of EEG datasets by recruiting a large enough group of participants is critical, as suggested in a recent review by Čukić et al. [8].

Indeed, developing a classification model that is resistant to large data variability due to highly diversified data sources is extremely challenging but critical. Aside from individual differences, data diversity can also result from differences in study designs, equipment and laboratory settings (EEG amplifier, room condition, etc.), EEG acquisition settings (reference electrode, sampling rate, etc.), and data collection procedures (preparation, instruction to participants, etc.) [28]. Any inconsistency in any factor across data collection sites will eventually result in higher inter-site data variability. Although it is possible to control for these factors across recording sites by applying rigorously standardized procedures (e.g., using the same equipment, experimental setting, and sequence of data collection), such control is not possible for a few other factors. For example, it is extremely difficult to maintain consistency in the recording environment (e.g., nature of lighting, room temperature, and ambient noise) consistent across hospitals. Another difficult factor to control is the technician’s skill, which is also a significant factor in signal variability. Assume one technician is very skilled in operating an EEG system (including applying electric gel to the electrodes) at one location, while another is qualified but not so skilled at another location. The time allotted for EEG cap preparation may differ between the two sites, and as a result, participants may potentially experience fatigue as a result of a longer and more uncomfortable preparation [28]. Even if the EEG recording is in a resting state, this will have an effect on the participants’ mental states. These inevitable factors of EEG variability have been considered to be a challenge to real-world brain-computer interface (BCI) [29,30] and EEG-based applications (e.g., seizure detection [31]). In this view, most of the previous EEG studies [9,10,11,12,13,14,15,16,17,18,19,20,21,22,23,24,25] have lacked a validation on datasets with high diversity (or large EEG variability) because their data were all collected from a single site.

### 1.3. Proposed Work

In summary, previous studies on EEG-based MDD detection using the ML approach suffer from the common limitation of small data size and diversity, which makes the effectiveness of the previously used EEG features or classification models in MDD detection still inconclusive (e.g., the frontal alpha asymmetry (FAA) [32]). To address this limitation, the present study launched a multi-site EEG data collection project. The large-scale EEG dataset used in this study contains the high-density (26 electrodes) resting-state EEG signals recorded from 400 age- and gender-matched participants (200 MDD and 200 HC from four different medical centers using the same recording protocols such as equipment, settings, and data collection procedures). As a result, the collected dataset has a relatively large data size and high diversity as compared with prior studies which, to the best of our knowledge, did not meet the two following criteria: (1) more than 300 participants and (2) more than two EEG recording locations.

A dataset with a large size and high diversity may result in a relatively large variation in the EEG data distribution. This could further make the MDD-HC classification problem even more challenging. To address this issue, selecting a set of discriminative EEG features and designing a robust ML classifier with improved generalization ability are crucial to detecting MDD with high sensitivity. Because this is the first study to use ML on a large EEG dataset, systematically re-examining the classification performance of commonly used EEG features is critical for proposing an effective and generalizable solution. To this end, in this study, we test the classification performance by using band powers (BP), coherence, and two types of fractal dimensions (FDs), i.e., Higuchi’s FD (HFD) and Katz’ FD (KFD). We investigate the performance of all possible BP and FD features at all electrodes and all frequency bands (we consider five different bands for BPs) for classifying MDD and HC groups. We also examine coherence features from all possible (a) regional inter-hemispheric, (b) cross-regional inter-hemispheric, and (c) intra-hemispheric electrode pairs at various frequency bands. This thorough analysis results in a total of 1859 features. Then, we used a wrapper-based feature selection approach, i.e., the sequential backward selection (SBS) algorithm [33], to find the optimal feature subset among all of the 1859 feature candidates for different classifiers, through which we can examine what combination of the commonly used EEG features would be the most sensitive to the detection of MDD.

Classifier design is also critical for the MDD detection performance. Previous EEG-based MDD detection studies have used a variety of classifiers, including the basic K-nearest neighbors (*K*-NN), linear discriminant analysis (LDA), logistic regression (LR), and the more advanced support vector machine (SVM). These classifiers have been extensively tested, and overall, the SVM consistently outperforms others [17,21,22,23]. However, when dealing with a large EEG dataset with a high degree signal variation, implying that the pattern distribution between the two classes of MDD and HC is likely to be highly non-separable, SVM’s performance might be comprised even if the optimal feature subset is used. To overcome this limitation, we use the conformal kernel (CK) technique [34] to improve the classification performance of the SVM. The success of CK-SVM has been shown in various EEG-based applications (e.g., [21,35]) that used relatively small EEG datasets. Because the CK transformation increases the spatial resolution around the SVM’s separation hyperplane in a kernel-induced feature space, we expect the CK-SVM can outperform the SVM on the larger-size and higher-diversity dataset used in this study.

Finally, we tested the proposed methods on an independent test set comprised of EEG data from 120 participants. We further systematically compare the performance of MDD detection using several popular but inconclusive EEG markers, with the goal of providing a thorough evaluation and reference of optimal EEG features for developing a reliable and effective EEG-based MDD-HC classification.

## 2. Cross Validation Used in Previous Works: More Detailed Review and Analysis

**Figure 1:**
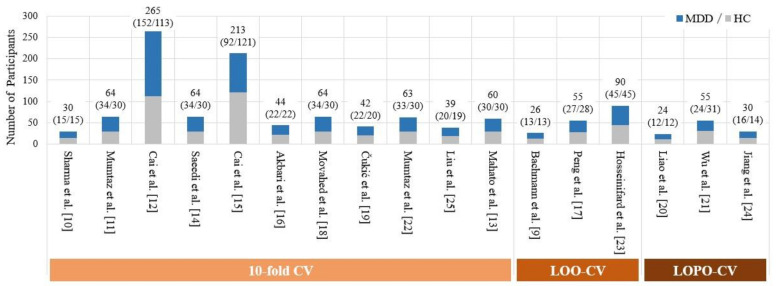
Comparison of the number of subjects in previous EEG- and ML-based studies.

Previous studies [9,10,11,12,13,14,15,16,17,18,19,20,21,22,23,24,25] had a very limited number of participants (see Figure 1 for detail). Therefore, it is difficult to partition the available EEG dataset of small sample size into a training dataset used for training classifiers and an independent test dataset of sufficient size. Due to this limitation, previous studies have only estimated the efficacy of their method in MDD detection by performing a k-fold CV on the entire EEG dataset, where k = 10 [10,11,12,13,14,15,16,18,19,22,25] or k = number of EEG samples [9,17,23] (i.e., leave-one-out (LOO)).

Directly performing a 10-fold CV on the group of participants (i.e., partitioning the entire group into 10 folds, with no EEGs of the same participants appearing in different folds at the same time) is nearly impossible due to participant size limitations. As a result, the k-fold CV is often performed on the EEG samples rather than participants. As an example, Movahed et al. [18] used a public EEG dataset (34 MDD, 30 HC) [36] and, for each participant, two EEG episodes of 5 min (eyes open and eyes closed) were collected. Their analysis procedure consisted of three steps: (1) data augmentation, in which the two signals of each participant were segmented into 10 EEG samples with 1 min lengths, resulting in a total of 6400 EEG samples at most; (2) data cleaning, in which noisy samples are removed; and (3) evaluation: by performing 10-fold CV on the remaining 510 samples (249 MDD and 261 HC EEG samples). They did not specify how many samples were saved from each participant after the second step. Nevertheless, it is reasonable to conclude that different samples of the same participant(s) were likely included in both training and test sets at the same time in each iteration of the 10-fold CV. Personal information leakage, which can be considered to be a type of data leakage known in the ML community [37], often results in the overfitting problem. As a result, such intra-participant EEG models often lead to higher classification performance, as emphasized in a recent review by Roy et al. [38].

To evaluate the participant-independent classification performance, a few studies used the leave-one-participant-out CV (LOPO-CV) strategy [20,21,24]. In each iteration of the LOPO-CV procedure, the EEG data of one participant were used for testing, while the data of remaining participants were used as the training set. Although the LOPO-CV avoids participant information leakage, another type of data leakage could occur. Take our previous studies [20,21] as examples, where the test data were actually used in the grid search-based feature selection and hyperparameter optimization. As a result, the test data in each iteration were not truly independent, but rather validation data. All these problems point to the same issue, i.e., a lack of an independent test set that meets the following two criteria: (1) containing a set of EEGs from a sufficiently large number of participants and (2) all these participants have never participated in the model training and validation.

## 3. Materials and Methods

### 3.1. Participants

**Figure 2:**
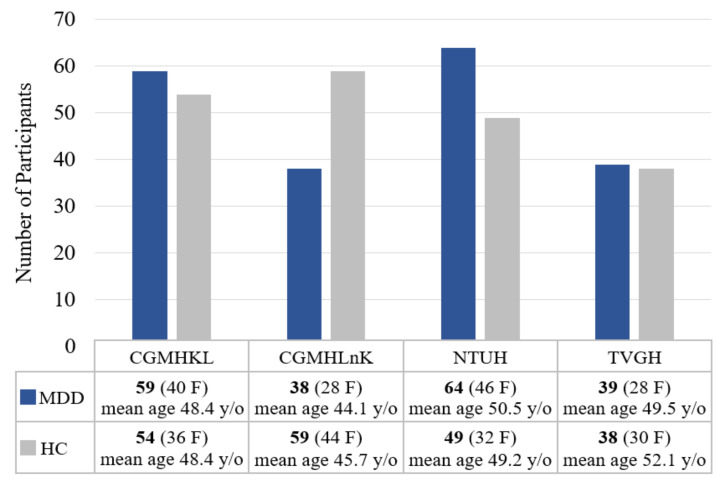
Gender distribution and mean age of participants from each medical center.

A total of 200 individuals with MDD (142 females, 52.85 years old on average and 58 males, 54.90 years old on average) and 200 age- and gender-matched healthy controls (142 females, mean age of 49.87 years and 58 males, 54.59 years old on average) were included in the study. Individuals with MDD were recruited from four tertiary medical centers: National Taiwan University Hospital (NTUH), Taipei Veterans General Hospital (TVGH), Chang-Gung Memorial Hospital KeeLung (CGMHKL), and Chang-Gung Memorial Hospital LinKou (CGMHLnK). Healthy controls were recruited from local communities surrounding each medical center. The current study included 64 MDD and 49 HC from NTUH, 39 MDD and 38 HC from TVGH, 59 MDD and 54 HC from CGMHKL, and 38 MDD and 59 HC from CGMHLnK. Figure 2 summarizes the gender distribution and mean age of participants from each medical center. All research protocols were reviewed and approved by the corresponding Research Ethic Committee from each medical center (IRB no: 201908038RSC for NTUH, 2019-09-004C for TVGH, and 201901342A3 for CGMH). All participants were informed of the study procedure and signed the consent forms before evaluations of medical history, family history, and neuropsychological tests.

The inclusion criteria for individuals with MDD were: (1) Age of 20 years or older, (2) with a diagnosis of major depressive disorder by two board-certified psychiatrists using the DSM-5 [39], (3) without any other psychiatry diagnoses based on evaluation of the Mini International Neuropsychiatric Interview (MINI) [40], and (4) the ability to comprehend the contents of consent forms. The inclusion criteria for the HC group were: (1) Age of 20 years or older, (2) no history of mental disorder as confirmed by a board-certified psychiatrist, and (3) capable of understanding the contents of consent forms. The general exclusion criteria for the current study were: (1) Confirmed pregnancy; (2) inability to understand the contents of consent form; (3) with a diagnosis of other types of mental disorder, including schizophrenia, psychotic disorder, bipolar disorder, obsessive and compulsive disorder, autism spectrum disorder, neurodevelopmental disorder, substance use disorder, neurocognitive disorder; (4) with a diagnosis of neurological disorder, including stroke, seizure, neurocognitive diseases, brain tumor; (5) a history of medication with anticonvulsant (e.g., Clonazepam) within 7 days before experiment; (6) severe vision or hearing impairment that leads to inability to perform cognitive tests; (7) a history of sustained loss of consciousness due to traumatic brain injury; (8) a history of other diseases that produce metabolic encephalopathy.

Each participant was interviewed by a psychiatrist for personal medical history, family medical history, and diagnosis. In addition, all participants completed the following questionnaires: Patient Health Questionnaire (PHQ-9) [41], Becks Depression Inventory–II (BDI-II) [42], and Hospital Anxiety and Depression Scale (HADS) [43]. Table 1 summarizes the demographic data and questionnaire scores for all participants. As can be observed, females were the gender majority on both MDD and HC groups. In many epidemiological studies, women were twice as likely to have depression as men [44]. The female predominance in our sample was mainly a result of gender difference in the epidemiological nature of MDD. Furthermore, since the gender distribution in the MDD and HC groups were similar, gender effect on electrophysiological biomarkers would not affect our findings.

### 3.2. Apparatus

**Figure 3:**
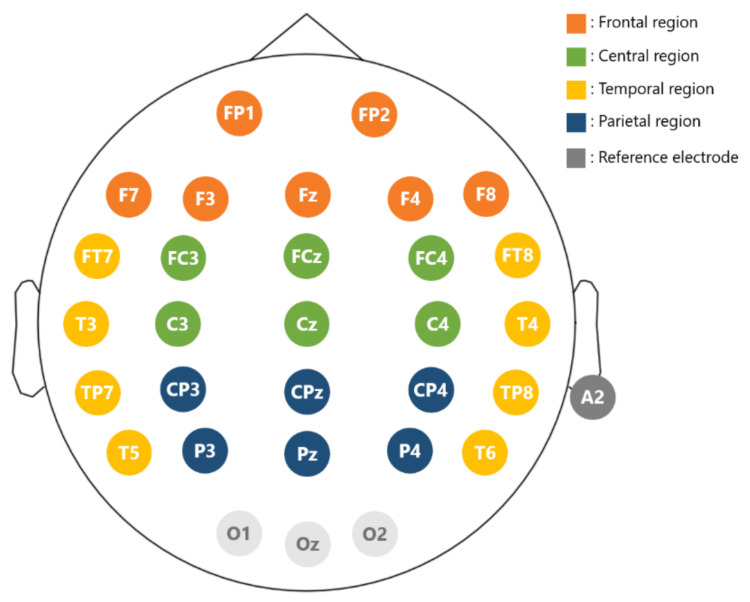
Electrode layout. Electrode positions follow the extended 10–20 international system. Reference is at A2. The entire scalp region is divided into four regions for analysis: frontal (brown), central (green), parietal (blue), and temporal (yellow).

Experimental stimuli were presented on a 24” flat screen monitor. Participants were sitting with their eyes 70 cm away from the monitor. The EEG data were recorded with a 32-channel Quick-cap (NeuroScan Inc., Charlotte, NC, USA) attached to four HNC amplifiers (HippoScreen Neurotech Corp.)with gain 50, A/D resolution 24 bit, and sampling rate 500 Hz). Electrodes were made of Al/AgCl material, and the montage was based on the extended international 10–20 system (Figure 3). Electrode gel (Quick-Gel by Compumedics Inc., Charlotte, NC, USA) was applied to the electrodes to keep the impedance below 10 K Ohm. Specifically for each electrode, we gently rubbed the surface of the scalp region while injecting gels so as to reduce the impedance level to be below the designated level of 10 k Ohm. EEG signals were on-line referenced to the A2 electrode (right mastoid).

### 3.3. Data Collection and Preprocessing

We recorded 90 s of eyes-open resting-state EEG signals from all participants. A timer was used to signal the start of the resting-state recording. Following the presentation of a fixation cross, participants were required to maintain a gentle fixation on the fixation cross for 90 s with their eyes open. During preprocessing, signals were re-referenced to vertex Cz and digitally filtered with a 2–50 Hz bandpass filter. Signals from O1, Oz, and O2 were excluded from analyses due to the constant signal instability caused by the cap fitness with Asian population (the back of heads of Asians are usually flatter and these electrodes were more often detached during recording). Then, an independent component analysis (ICA) conducted based on the Picard algorithm [45] was used to remove artifacts caused by eye movements and eye blinks [46], which was implemented with MNE-Python. Then, the 90 s resting-state EEG data from the remaining 26 electrodes were divided into 15 non-overlapped 6 s EEG epochs.

### 3.4. Feature Extraction

#### 3.4.1. Band Power (BP)

The power spectrum density (PSD) of each of the 15 6 s EEG epochs from the same electrode was computed using a Fast Fourier Transform (FFT), and the powers of delta (2–4 Hz), theta (4–8 Hz), alpha (8–13 Hz), beta (13–30 Hz), and gamma (30–45 Hz) frequency bands were calculated separately. Then, the 15 band powers (BPs) of the same electrode’s 15 EEG epochs were averaged for each band. Given the very large values of the calculated BP values, we took the logarithm to the averaged BP value (a log BP). We obtained a total of 130 log BP features from each participant’s EEG because there were five frequency bands and 26 electrodes. The BPs in the following refer to the log BPs. It should be noted that the delta band used in this study is narrower than the commonly used 0.5–4 Hz. As a result, the delta band in this study refers to the fast delta [47].

#### 3.4.2. Coherence

EEG coherence was also calculated by the FFT method. For each pair of electrodes and for each band f, the squared band coherence value between two EEG epochs *i* and *j* was calculated spectrally as:

(1) $$COH_{ij}^{2}\left(f\right)=\frac{{\left|G_{ij}\left(f\right)\right|}^{2}}{G_{ii}\left(f\right)G_{jj}\left(f\right)}\;$$

where $COH_{ij}\left(f\right)$ is the coherence on band f, $G_{ij}\left(f\right)$ is the averaged cross-power density, and $G_{ii}\left(f\right)$ and $G_{jj}\left(f\right)$ are respective averaged auto-power spectral densities across the frequency band f. The coherence value lies in the interval $\left[0,\;1\right]$. A coherence value that is close to 1 corresponds to a strong linear association between the EEG signals. Then, we computed the averaged coherence values across 15 epochs (referred to as coherence hereafter). We extracted a total of 1625 coherence features from each participant’s EEG (26 × 25/2 electrode pairs × 5 bands).

#### 3.4.3. Higuchi’s Fractal Dimension (HFD)

Fractal dimensions (FD) have been used to measure complexity of a time series. The FD of a time series can be estimated by time domain or phase space domain approaches [48]. The former estimates the FD directly in the waveform of a signal, such as the Higuchi and Katz’s methods used in the current study. The latter estimates the FD of an attractor in the state-space domain. Compared to the phase space domain approach, the time-domain approach has the advantage of fast computation.

In this study, each EEG epoch is a 6-second signal containing 3000 sample points (sampling rate = 500 Hz). For such a short-time segment, HFD has been shown to be a suitable FD estimator [49,50]. We first reconstruct *k* new signals from a time series of *N_s_* samples (*X* = [*X*(1), *X*(2), …, *X*(*N_s_*)] as:

(2) $$X_{m}^{k}=\left[X\left(m\right),X\left(m+k\right),X\left(m+2k\right),\ldots ,\;X\left(m+int\left(\frac{N_{s}-m}{k}\right)k\right)\right],\;m=1,2,\ldots ,k\;$$

where *m* is the initial time point, *k* is the time interval, and *int*() denotes integer function. Here, we reset the definition of the symbol *k* from the number of folds in a CV procedure to the number of reconstructed signals, in order to be consistent with the symbol used in previous HFD studies. For a starting time point *m*, the length $L_{m}\left(k\right)$ of the corresponding curve (i.e., reconstructed signal) $y_{m}^{r}$ is given by:

(3) $$L_{m}\left(k\right)=\frac{1}{k}\left[\frac{N_{s}-1}{int\left(\frac{N_{s}-m}{k}\right)k}\left(\overset{int\left(\frac{N_{s}-m}{k}\right)}{\underset{i=1}{\sum }}\left|X\left(m+ik\right)-X\left(m+\left(i-1\right)k\right)\right|\right)\right]\;$$

which represents the normalized sum of absolute values of differences between two consecutive points separated by a distance of *k*. Foreach time interval *k,* the average curve length is then calculated by:

(4) $$L\left(k\right)=\frac{1}{k}\overset{k}{\underset{m=1}{\sum }}L_{m}\left(k\right)\;$$

The HFD value of the time series is the slope of *log*(*L*(*k*)) against *log*(1/*k*) within the range of *k* = 1, 2, …, *k*max, which can be calculated using a least-squares linear fitting. 

The HFD value lies within the range between 1 and 2. The HFD value is equal to 1 for a deterministic signal, and a signal with a higher HFD value corresponds to a higher irregularity. The HFD calculation involves one free parameter, the maximum value of *k*, *k*max. It has been suggested in a motor imagery study [51] that the setting of *k*max = 100 is appropriate for the detection of change in brain dynamics. Here, we tried three different settings (*k*max = 50, 100, and 150) to seek for a better solution of the HFD for MDD-HC classification. In the present study, for each electrode, we calculated the HFD value of each EEG epoch, and then computed the averaged HFD values across 15 epochs. Finally, a total of 78 HFD features were extracted from each participant (26 electrodes × 3 parameter values). 

#### 3.4.4. Katz’s Fractal Dimension (KFD) 

Katz’s method [52] estimates the FD of a time series directly from its waveform by

(5) $$\frac{log\left(\frac{L_{w}}{A}\right)}{log\left(\frac{D_{w}}{L_{w}}\right)+log\left(\frac{L_{w}}{A}\right)},\;$$

where $L_{w}$ and $D_{w}$ are the total length and the planar extent of the waveform, respectively, and A is the average of the distances between two successive sample points of the time series. The value of KFD also lies within the range between 1 and 2. The complexity of a time series increases with the KFD value. In this study, the KFDs of the 15 EEG epochs from the same electrode were calculated, and then averaged. Therefore, we obtained a total of 26 KFD features from each participant.

In total, we extracted a total of 1859 features (130 BPs + 1625 coherences + 78 HFDs + 26 KFDs) from each participant’s 90 s long, 26-channel EEG signals. In addition, since we used a feature averaging strategy across epochs in the feature extraction process, there is only one value for each feature eventually. As a result, for example, there is only one 26-dimensional feature vector (also known as a data or a data point in the following) is extracted from each participant if all of the alpha BP features from the 26 electrodes are used for classification.

### 3.5. Classification

Four different classifiers were compared: K-NN, LDA, SVM, and CK-SVM. Here, we briefly introduce the LDA and SVM, and then show how the conformal kernel transformation improves the conventional SVM’s performance, i.e., CK-SVM.

#### 3.5.1. K-NN

K-NN is an instance-based learning method. A test data point is compared to K-nearest data points in the training set. Then, the class label of the test data point is assigned according to majority voting among the classes of the K-nearest training data points. The Euclidean metric is used to calculate the distance between data points in this study.

#### 3.5.2. LDA

Suppose that n features are used for classification. The *LDA* classifier finds a hyperplane $w_{lda}^{t}x+b_{lda}=0\;$to separate the two classes of *MDD* and *HC* in the original space of patterns $R^{n}$ [21], where:

(6) $$w_{lda}={\left(m_{MDD}-m_{HC}\right)}^{t}\Sigma^{-1}\;$$

is the weight vector.

(7) $$b_{lda}=\frac{1}{2}{\left(m_{MDD}-m_{HC}\right)}^{t}\Sigma^{-1}\left(m_{MDD}+m_{HC}\right)-ln\left(\frac{C_{MDD}p_{HC}}{C_{HC}p_{MDD}}\right)\;$$

is the bias of the separating hyperplane; $m_{MDD}\;$and $m_{HC}$ are the mean vectors of the training data of *MDD* and *HC* classes, respectively; Σ is the covariance matrix of the set containing both classes’ training data; $C_{MDD}$ and $C_{HC}\;$are penalties assigned to the MDD and HC classes, respectively; and $p_{HC}\;$and $p_{MDD}\;$are the a priori probabilities of the HC and the MDD classes, respectively. An unseen data $x\in R^{n}$ is classified as MDD if $D_{LDA}\left(x\right)=w_{lda}^{t}x+b_{lda}>0$; otherwise, it is classified as HC. 

#### 3.5.3. SVM

An SVM maps the training data $x_{i}\in R^{n}$, i=1,…, M, into a higher dimensional feature space F via a nonlinear mapping $\varphi :R^{n}\to F$, and then finds an optimal separating hyperplane $H=w^{t}\varphi \left(x\right)+b=0$ that maximizes the margin of separation and minimizes the training error. The decision function of SVM is given by:

(8) $$D_{SVM}\left(x\right)=\underset{x_{i\;}\in SV}{\sum }\alpha_{i}y_{i}K\left(x_{i,\;},x\right)+b\;$$

where $0\leq \alpha_{i}\leq C\;$are Lagrange multipliers; $y_{i}\in \left[-1,+1\right]\;$are class labels of the training data; C is the penalty weight that needs to be determined experimentally; SV stands for the support vectors, i.e., the training data whose $\alpha_{i}\prime s\;$satisfying the condition $0<\alpha_{i}\leq C$; the bias b is computed using the Kuhn–Tucker conditions of SVM; and $K\left(,\right)\;$is the kernel function that computes the inner product of two mapped data points. Here, we adopted the Gaussian kernel defined by $K\left(x_{i,\;},x_{j\;}\right)=exp\left(-\gamma ∥x_{i}-x_{j\;}∥{}^{2}\right)$, where γ is the kernel parameter. The class label of an unseen data x is predicted as MDD if $D_{SVM}\left(x\right)>0$; otherwise x is classified as the HC group.

#### 3.5.4. CK-SVM

A Gaussian kernel-induced feature space F is of infinite dimension, and the Gaussian kernel embeds the original space $R^{n}\;$as a Riemannian manifold distributed on the unit sphere centered at the origin of the space F (please refer to the author’s study [53] for a detailed explanation). Thus, the relationship between a small shift of the Riemannian distance ds and a small displacement in the original space dx can be described by the Riemannian metric $g_{ij}\left(x\right)\;$as follows:

(9) $$ds^{2}=\underset{i,j}{\sum }g_{ij}\left(x\right)dx_{i}dx_{j}=\underset{i,j}{\sum }{\left.\frac{\partial K\left(x,x^{\prime }\right)}{\partial x_{i}\partial x_{j}^{\prime }}\right|}_{x^{\prime }=x}dx_{i}dx_{j}\;$$

where $x_{i}$ and $x_{j}$ denote the ith and jth elements of the vector x, respectively. This shows that a local volume’s size in $R^{n}\;$can be enlarged or shrink in F by conformally transfroming the mapping as $\tilde{\varphi }\left(x\right)=c\left(x\right)\varphi \left(x\right)$ and choosing a proper conformal function as [34]:

(10) $$c\left(x\right)=\underset{x_{i}\in T}{\sum }exp\left(\frac{-∥\varphi \left(x\right)-\varphi \left(x_{i}\right){∥}^{2}}{\frac{1}{n_{e}}\sum_{j}∥\varphi \left(x_{j}\right)-\varphi \left(x_{i}\right){∥}^{2}}\right)\;$$

where the term $\sum_{j\neq i}∥\varphi \left(x_{j}\right)-\varphi \left(x_{i}\right){∥}^{2}\;$/$n_{e}$ is the average squared distance from the mapped data point $\varphi \left(x_{i}\right)$ to its $n_{e}\;$nearest neighbors $\varphi \left(x_{i}\right)$. Notice that the expression of Equation (10) can be further simplified by kernel tricks, and $K\left(x_{i,\;},x_{i,\;}\right)=1,\;\forall i$ in the present study, because of the use of Gaussian kernel. The conformal function decreases exponentially with the distance from $\varphi \left(x\right)\;$to the image of the set *T*. By this setting, the spatial resolution is enlarged around the images of $x_{i}$ and contracted elsewhere in the feature space of F. The inner product of two conformally transformed data points $\tilde{\varphi }\left(x\right)$ and $\tilde{\varphi }\left(x^{\prime }\right)$ further leads to the form of a conformal transform of a kernel:

(11) $$\tilde{K}\left(x,x^{\prime }\right)=c\left(x\right)c\left(x^{\prime }\right)K\left(x,x^{\prime }\right)\;$$

where $\tilde{K}$ is the so-called conformal kernel (CK).

The design of the function $c\left(x\right)\;$is cricual to the performance of the CK method. In the original work by Wu and Amari [34], they proposed that the set T should include the support vectors, based on the assumption that most support vectors within the margin of separation. By doing so, increasing the spatial resolution around the mapped support vectors is equivalent to enlarging the spatial resolution of the margin of separation in the feature space F. This leads to a better class separability, further resulting in increased generalization. However, in a highly non-separable case, many support vectors would fall outside the margin rather than within it. To remedy this problem, Wu et al. [21] proposed that the set T should only include the support vectors within the margin (SVWM):

(12) $$\mathrm{SVWM}=\{x_{i}|-1\leq w^{t}\varphi \left(x_{i}\right)+b\leq 1\}\;$$

and that the number of nearest neighbors in the set T can be set as $n_{e}=3$. 

In the current study, we use this strategy to increase the spatial resolution around the images of the SVWM to further increase the spatial resolution in the vicinity of the SVM’s separating hyperplane in the feature space. However, we set $n_{e}=5$, for the reason that it is expected that the SVWM would be much more in the current study, because of the use of a much larger and more diverse EEG dataset. In summary, the CK-SVM has two training steps,

**Step 1** Train the SVM with a training set and a chosen kernel $K\left(,\right)$, and determine the optimal hyperparameter.

**Step 2** Use the conformal kernel $\tilde{K}\left(,\right)$ calculated by Equation (11) to retrain the SVM with the same training set and the same optimal hyperparameter of the SVM.

It should be noted that using the same training set in Step 2 means that the features are also the same as those used in the training set used for the SVM in Step 1. Finally, the retrained SVM is called CK-SVM.

### 3.6. Hyperparameter Optimization Procedure

For the K-NN classifier, we set K=3. In LDA, we assigned the same penalties to both MDD and HC classes by setting $C_{MDD}/C_{HC}=1$. The SVM involves two free parameters, i.e., the penalty weight C and the kernel parameter γ. The two parameters were optimized using a 5-fold CV procedure combined with a grid search. Initially, we searched for the suboptimal value of γ within a wide range with a rough grid, and then searched the optimal solution with smaller parameter grids in the set $\left\{\left(C,\gamma \right)|C=1000,\;10000;\;\gamma =0.0001:0.0001:0.01\right\}$ containing a total of 200 girds. Given a training set S, the entire hyperparameter optimization consists of two steps: (1) For every grid, the 5-fold CV is performed once on S and (2) the optimal grid is the one giving the highest 5-fold CV classification accuracy.

### 3.7. Determine the Optimal Feature Subset Using Sequential Backward Selection

The total number of features in the current study is quite large. The best combination of features for classification must be determined. In this case, wrapper methods are preferable to filter methods (e.g., ranking each feature by Fisher’s criterion [54]) for feature selection. The wrapper approach is classifier specific, which determines the optimal feature combination directly by using the generalization performance (e.g., cross-validation accuracy) as the evaluation criterion. As a result, the wrapper approach often outperforms the filter approach in terms of feature selection [55]. Therefore, we adopted a popular wrapper method, i.e., the sequential backward selection (SBS) [33], to select the optimal feature set from the 1859 candidates.

SBS is a heuristic search method. Let *N* represent the number of feature candidates. Initially, a 5-fold CV is performed on the dataset with all the *N* features. Following that, each feature is removed, one at a time. The 5-fold CV is applied to all *N*-1 features subsets. Then, the worst feature is discarded. Here, the worst feature means that without it, the subset with the remaining *N*-1 features gives the highest cross-validation accuracy among all the *N*-1 subsets. In the next round, each of the *N*-1 features is deleted one at a time, and the worst feature is discarded, using the same logic. A subset with *N*-2 features is formed. This procedure is repeated until none of the *N* features is left. Finally, the optimal feature subset is the one that produces the highest 5-fold CV accuracy. It should be noted that if the chosen classifier is SVM, the SBS-based feature selection must be performed together with the classifier’s hyperparameter optimization procedure.

## 4. Results

To provide a reliable result with high generalizability, the collected dataset (Table 1) was divided into a training set and a test set. The training set included the EEG data of 280 participants (140 MDD and 140 HC), while the test set included the EEG data of the remaining 120 participants (60 MDD and 60 HC). The training set was used for feature selection as well as classifier training and validation. The test set was used to see how well the trained and optimized methods generalized to another independent group of 120 participants. Table 2 and Table 3 summarize the demographic data of the training and the test sets, respectively. 

### 4.1. Electrode- and Region-Specific Comparison of 5-Fold CV Classification Accuracy between Features Using LDA Classifier

Here, we compare the 5-fold CV-based MDD-HC classification accuracy (referred to simply as accuracy in this subsection) between commonly used features in terms of different scalp regions (region level) and different electrodes (electrode level). Prior to using a nonlinear classifier such as SVM, first, we use an LDA classifier to examine whether the features in different scalp regions can be well separated by the linear decision boundary learned from LDA to examine the linear class separability of the features in different electrodes and scalp regions.

Here, we use the standard accuracy as the performance metric:

$$Accuracy=\left(TP+TN\right)/\left(TP+FN+TN+FP\right)$$

where *TP*, *TN*, *FP*, and *FN* represent true positive, true negative, false positive, and false negative, respectively. Because both the training and test sets have equal numbers of MDD and HC data, the classification accuracy would not result in biased classifier performance. Moreover, because the dataset is balanced, the accuracy identical to the average of sensitivity (true positive rate) and specificity (true negative rate), which are two measures in clinical evaluation of medical devices.

**Figure 4:**
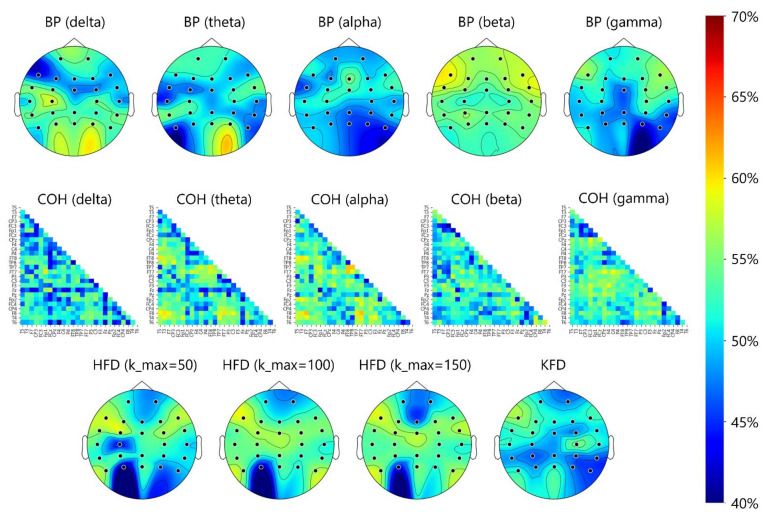
Topoplots of 5-fold CV classification accuracies in BP, coherence (COH), HFD, and KFD features. The accuracy distribution shown around the occipital area should be ignored, because the EEGs from O1, O2, and Oz were not analyzed in the present study.

Figure 4 shows the topoplots that depict the electrode-specific results. Overall, for all bands, the classification accuracy based on each electrode’s BP feature was below 60%. The HFD and KFD features yielded the same results. The best accuracy for a single electrode was 59.29% (beta BP at F7). For coherence, a few electrode pairs provided accuracies slightly higher than 60%. The best accuracy of 61.71% was obtained from the alpha-band coherence feature of the F8-FT7 pair, which is only slightly higher than the chance level of 50%. Table 4 shows the scalp-region-specific results. Take the beta BP of the frontal region as an example, the seven beta-band BP features from seven frontal electrodes (Figure 3) were all fed into the LDA classifier without feature selection and the accuracy was 56.07%. In the same frontal region, the accuracy increased slightly (61.07%) when the best two beta-band BP features (FP1 and FP2) determined by the SFS algorithm. The results under the “ALL” category were based on features extracted from the 26 electrodes or 26 × 25/2 electrode pairs in the frontal, central, parietal, and temporal regions. 

Table 4 shows that in the condition of “without feature selection”, all the combinations of features and regions do not show any discriminative ability (<60%). For most of the combinations, the accuracy improves after feature selection. However, the improvement is limited (slightly greater than 60%) for all intra-regional and the ALL-regional features, with the exception of the ALL-scalp coherence. The results show that after feature selection, the accuracy of using the coherence features was greater than 80% for all bands except for the beta coherence features. On the one hand, a set of optimal theta-band coherence features extracted from the entire scalp’s electrode pairs, in particular, achieved the highest accuracy of 83.21%. BP and FD (including KFD and HFD) features, on the other hand, did not perform well even when using their corresponding optimal feature subsets for each scalp region.

### 4.2. Feature Fusion and Feature Selection Results

**Figure 5:**
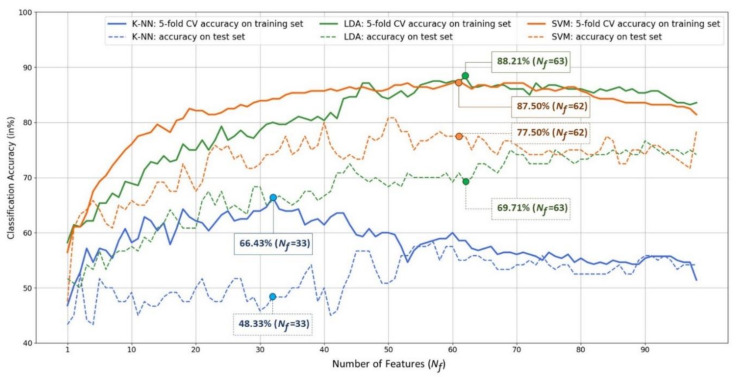
Plots of classification accuracy (vertical) versus the number of features determined by the SBS-based feature selection procedure (horizontal). The solid curves represent the MDD-HC classification accuracy obtained by performing the 5-fold cross validation on the training set using different classifiers. The dotted curves represent the classification accuracy on the test set.

Next, we ran the SBS feature selection on all 1859 feature candidates composed of all BP, coherence, HFD, and KFD features, to examine if the feature fusion and selection strategy could achieve high 5-fold CV accuracy on the training set and satisfactory accuracy on the test set. This procedure was performed separately for the three classifiers, i.e., *K*-NN, LDA, and SVM, because this wrapper approach of SBS is classifier specific. Figure 5 shows the results. For each classifier, a 5-fold CV classification accuracy was obtained for a given number of features. As a result, the range of the number of features, $N_{f},$ was supposed to be between 1 and 1859. However, it would be unnecessary to show the change in the accuracy with respect to all possible numbers of features from 1859 to 1, because, for the three classifiers, their corresponding numbers of best features were all less than 70. As a result, in Figure 5, we only show the accuracy curves within the range of $N_{f}\in \left[1,\;99\right]$, so that the subtle variation of accuracy within this range can be seen more clearly. Note that the number of features $N_{f}$ is in fact identical to the dimension n of an input data described in Section 3.5.2 (LDA) and Section 3.5.3 (SVM).

**Figure 6:**
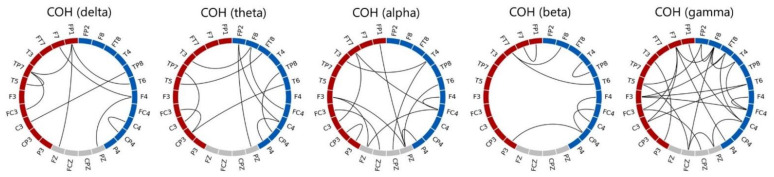
Plots of the 61 optimal coherence features of SVM determined by the SBS-based feature selection procedure. Electrodes at the left and right hemispheres are marked in red and blue, respectively. It is noted that the line connected between an electrode pair does not represent that the coherence between the two electrodes is higher than a threshold, but shows that the coherence feature of the electrode pair is one of the 61 optimal coherence features.

**Figure A1:**
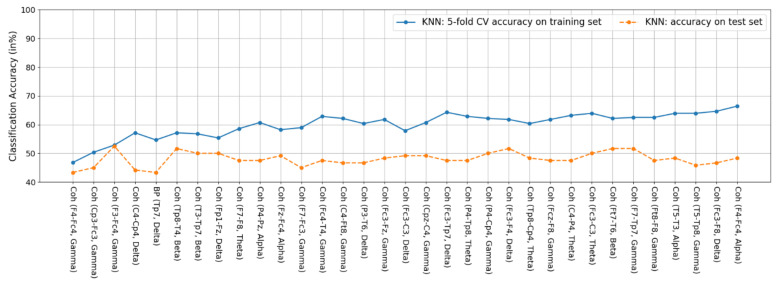
Features of the *K*-NN classifier. Among the 33 features, 32 are coherence features and the remaining one feature is the delta BP feature at TP7.

**Figure A2:**
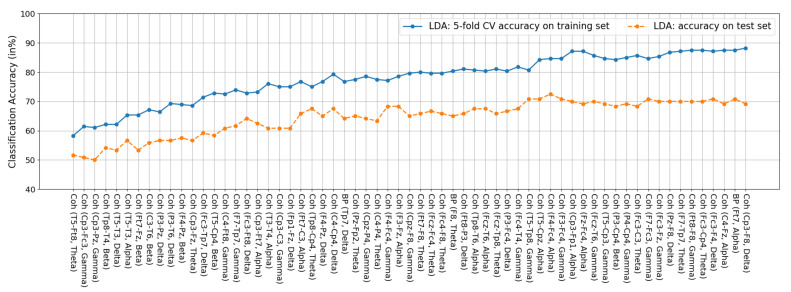
Features of the LDA classifier. Among the 63 features, 60 are coherence features and the remaining three are all BP features.

**Figure A3:**
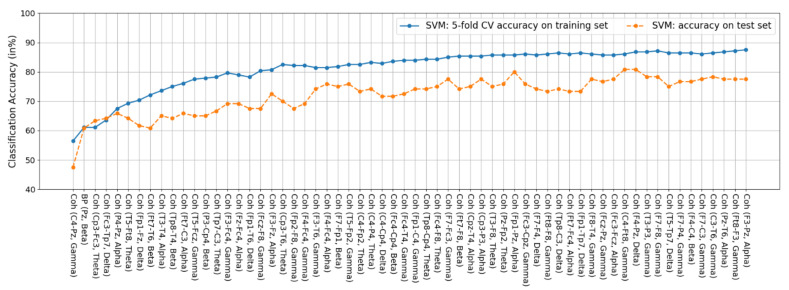
Features of the SVM classifier. Among the 62 features, 61 are coherence features and the remaining one is the beta BP feature at Pz.

There are four observations shown in Figure 5. (1) In terms of 5-fold CV classification accuracy performed on the training set, the best accuracy of the K-NN, LDA, and SVM classifiers are 66.43%, 88.21%, and 87.50%, respectively. Furthermore, the best numbers of features for the three classifiers are 33, 63, and 62, respectively. LDA achieved the highest CV classification accuracy. (2) However, when applying the trained models (the classifiers trained with the corresponding optimal feature subsets) to the test set of 120 independent participants (each misclassified participant results in a 0.83% = 100/120 accuracy drop), on the one hand, the LDA classifier’s performance dropped significantly from 88.21% to 69.71%, indicating poor generalization. SVM, on the other hand, outperformed the others and can maintain an MDD-HC classification accuracy close to 80% (77.5%) on the test set. K-NN performed the worst of the three classifiers, with a testing accuracy of 48.33%. (3) The feature subset achieving the highest 5-fold SV classification accuracy does not necessarily guarantee the best performance when confronted with unseen data. For example, as $N_{f}=51$, although SVM’s 5-fold CV training accuracy of 86.07% was not the best, it achieved the highest classification accuracy of 80.83% on the unseen test set (i.e., 97 out of the 120 participants in the test set were correctly classified). (4) According to the 5-fold CV accuracy on the training set, the SVM’s optimal feature subset determined by the SBS search consists of 62 features, which are all coherence features except one beta-band BP feature of the Pz electrode. Figure 6 depicts the 61 optimal coherence features in various bands, suggesting that the functional connectivity in all bands is critical in classifying MDD and HC groups when the SVM is used as a classifier. Furthermore, coherence features make up the majorities of the 33 and 63 optimal features for *K*-NN and LDA classifiers (see Figure A1, Figure A2, and Figure A3 for a detailed feature performance ranking of the optimal features for the three classifiers in Appendix A). Table 5 shows the numbers and the percentages of BP, COH, HFD, and KFD in the optimal feature subset for each classifier. It can be concluded, here, that the coherence features contribute the most to the highest 5-fold CV performances for all the three classifiers.

In addition, Figure 5 shows that there is a performance drop on the test set. The 5-fold CV was used in conjunction with feature selection (for all three classifiers) and the hyperparameter optimization of SVM classifier. Therefore, the data leakage described in Section 2 did occur, leading to overfitting. It is, however, unavoidable. Fortunately, the highest accuracy of SVM classifier on the test set achieve 80.83%.

### 4.3. Comparison of Classification Accuracy between the CK-SVM and Other Classifiers

This section compares the classification performance of CK-SVM with that of *K*-NN, LDA, and SVM in terms of both the 5-fold CV and testing classification accuracies. For comparison, the results of two SVM in the cases of $N_{f}=51$ and $N_{f}=62$ shown in Figure 5 were used, because the former achieved the best classification accuracy on the test set and the latter achieved the best 5-fold CV accuracy on the training set. The optimal SVM hyperparameters $\left(C,\;\gamma \right)$ for the two cases are $\left(10000,\;0.0004\right)$ and $\left(10000,\;0.0003\right)$, respectively. It is worth noting that CK-SVM does not need to find its optimal hyperparameter with a grid search again. As long as the optimal hyperparameters of SVM are found, we can, then, retrain SVM with a conformal kernel using the same parameters and the same features. As a result, the same hyperparameters were used to train the CK-SVMs in the cases of $N_{f}=51$ and $N_{f}=62$.

Table 6 summarizes the classification accuracies of the 5-fold CV and testing procedures for various classifiers. First, the CK-SVM performed the best among all the classifiers, while the *K*-NN classifier performed the worst. The highest CV classification accuracy of 91.07% was achieved by the CK-SVM when its 51 optimal features were used. Using this optimal subset, it also achieved the best MDD-HC classification accuracy of 84.16% on the test set. In other words, 101 out of the 120 participants in the test set were correctly classified by the CK-SVM, with the sensitivity 88.33% (53 out of the 60 testing MDD participants were successfully detected) and specificity of 80% (48 out of the 60 HC participants were correctly classified). Second, with both training and testing sets, the CK-SVM outperformed conventional SVM in the MDD-HC classification. For example, in the case of 5-fold CV performed on the training set, the CK-SVM outperformed the SVM in both conditions of $N_{f}=51$ and $N_{f}=62$ by 5% (91.07–86.07%) and 1.78% (89.28–87.50%), respectively.

### 4.4. Comparison with Previous Literature: Statistical Analysis on Frontal Alpha Asymmetry (FAA) and HFD Complexity

**Figure 7:**
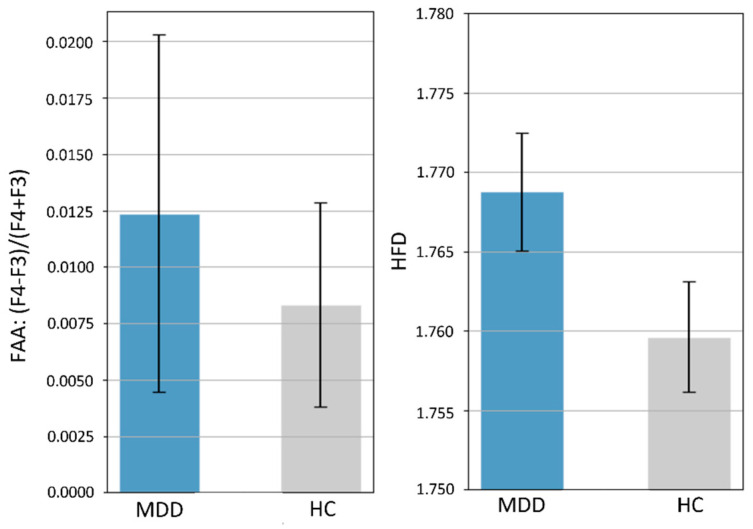
Means and 95% error bars (standard error of the mean) for MDD and HC participants. Left: frontal alpha asymmetry (FAA) calculated by the ratio (F4 − F3)/(F4 + F3) of alpha power. Right: EEG complexity represented by the HFD with *k*max = 50. There were no significant differences in FAA (*p* = 0.66) and HFD-based complexity (*p* = 0.07) between the MDD and HC groups.

Finally, we compared our results to previous research using two commonly used features: the frontal alpha asymmetry (FAA) and HFD complexity. Specifically, we used a Student’s t-test to statistically test for group differences in FAA and HFD complexity of EEG from all electrodes (mean of 26 electrodes’ HFD) on the 400 participants. Figure 7 shows that the average FAA in the MDD group (0.0124 ± 0.1123) was higher than that in the HC group (0.0083 ± 0.064), but the difference was not statistically significant (*p* = 0.66). There was also no significant difference in HFD-based EEG complexity between groups (MDD, 1.7688 ± 0.0522 and HC: 1.7596, *p* = 0.07).

## 5. Discussion

In this section, we compare our results with previous studies in terms of the four EEG markers: FAA, HFD-based complexity, BP, and coherence features. It should be noted that a direct comparison between previous findings and ours could be a bit unfair, because there are many differences in terms of EEG protocols and, particularly, the nature of EEG datasets. Nevertheless, such comparison can still provide important insight into some worthwhile discussion issues.

*FAA*. Several studies have suggested that the FAA of resting-state EEG could be a neurosignature for the diagnosis of MDD. For example, Debener et al., 2000 (15 MDD, 22 HC) [56] and Roh et al., 2020 (67 MDD, 60 HC) [57] reported a significant difference in FAA between the MDD and HC groups. Our result is inconsistent with theirs, but is consistent with the findings of Knott et al., 2001 (69 MDD, 23 HC) [32], Vinne et al., 2017 (1008 MDD, 336 HC) [58], and Smith et al., 2018 (143 MDD, 163 HC) [59]. However, some variables may influence the FAA, such as the severity of depression. In the study by Roh et al. [57], 23 of the 67 patients with MDD had suicidal ideation, which has been shown to be a clinically important moderator of FAA in patients with MDD.

*Complexity of EEG Activity*. There is substantial evidence showing that increased complexity of resting-state EEG activity can be measured in MDD (e.g., [23,60,61], see [62] for a review on the EEG complexity in MDD detection). In addition, the EEG HFD complexity has also been shown to be capable of producing a high MDD-HC classification [61]. Although our results also show increased HFD values of individuals with MDD as compared with HC (no statistical significance), the HFD feature did not provide satisfactory classification performance (maximum 62.86% based on LDA, see Table 4) in the current study. This low and inconsistent (as compared with previous works) accuracy level could be due to a number of factors. One possible factor is the condition of data collection; our EEG data were collected during the eye-open resting-state, whereas previous studies [23,61] collected EEGs during the eye-closed resting-state. For example, using HFD as features, a study by Hosseinifard et al. [23] showed a LOO-CV accuracy of 73.3% with the LDA classifier, whereas a study by Čukić et al. [61] showed an average accuracy of 89.90% across different classifiers (multilayer perception, logistic regression, SVM, decision tree, random forest, and Naïve Bayes). Other factors such as differences in the EEG amplifiers (dB, gain, and input noise specifications) and the signal preprocessing methods (band-pass filtering, artifact removal, signal epoch length, etc.) may also affect the signal-to-noise ratio of EEG signals. As a result, due to these possible factors and the fact that HFD is sensitive to noise [48], the effectiveness of HFD in detecting MDD may differ. More importantly, the current study collected EEG data from four different medical centers, whereas previous studies only collected EEG data from a single location. The high diversity of the current EEG dataset may result in highly variable noise levels.

*BP*. A few previous studies have suggested that alpha BP could be an MDD indicator. Our previous work [20] showed a nearly 70% (68.98%) classification accuracy using all-electrode alpha BP features and a *K*-NN classifier. However, in the current study, the all-electrode alpha BP (without feature selection) could only achieve an accuracy of 47.50% (Table 4). Another study applied an LDA-based classifier to the alpha BP features at C3, P3, O1, O2, and F7 [23] and reported a classification accuracy of 73%. In our study, the accuracy of the alpha BP remained below 60% (58.21%) even after feature selection. A discrepancy in alpha BP’s ability to classify appears to exist between studies. The empirical results of this study suggested that including the BP feature in the EEG protocols alone may not be an accurate and reliable way to detect MDD.

Intra-participant variability in EEG amplitudes could contribute to the inconsistent results in the classification performance of BP. Often, every EEG study would keep the impendence of every electrode below a desired level before recordings. However, in practice, maintaining impendence stability throughout the recording period is quite challenging. The slight loss of an electrode could cause the impendence to change, resulting in changes in EEG amplitudes. Note that in some studies, noisy EEG segments were often removed from analyses (e.g., [18]). In the current study, however, we did not adopt this data cleaning strategy because, for the current study, an important objective was to verify the effectiveness of a feature in MDD detection, and also the robustness of a feature that could overcome the technical instability that may occur in real-world applications. Moreover, maintaining a stable impedance/signal quality across multiple recording sites is even more challenging [28]. As a result, both intra-subject and inter-site EEG variabilities could have contributed to the poor performance of the BP feature.

In the experiments, we did not perform any EEG normalization because, as previously noted, the aim was to test the performance of the commonly used EEG features in previous MDD studies and, for example, the band powers in those studies are absolute powers without any normalization. However, it is worthwhile to investigate whether performing an EEG normalization can affect the BP performance. To this end, we normalize the power of each channel by subtracting the average power of the 26 channels in each frequency band, which can be viewed as a BP-based common average referencing (CAR). Table 7 summarizes the findings. As compared with the BP performance listed in Table 4 (the best performance is 64.64% without normalization), the normalized BP achieved comparable performance (the best is 63.57%). This comparison shows that there is almost no difference after using the CAR normalization method. One possible reason is as follows.

The topographical BP distribution of EEG signals are mainly determined by the relative differences in BP between channels. Although the BP-based CAR normalization may reduce the variability caused by possible baseline BP differences between subjects, it does not affect the EEG BP distribution over the scalp for each individual because the average power is subtracted equally from all channels. This was nicely depicted as the effect of the rising or receding water levels of a lake in a mountainous area, which changed the location of the zero water level mark, but not the landscape [63]. For example, in the situation where one electrode’s impendence changes during the EEG recording process, the BP will still vary even if such normalization is performed afterwards. Unfortunately, this happens in real-world applications, as mentioned above.

*Coherence*. The coherence (more precisely, the spectral coherence) has long been proposed as a fundamental mechanism for communication within brains [64] and is not affected by the amplitude changes of EEG oscillations [65], and therefore this property plays a key role in improving classification in the dataset with large intra-participant and inter-site EEG variability [66]. This could explain why 61 of the 62 optimal features selected from all 1859 feature candidates are all coherence features in the current study.

**Figure 8:**
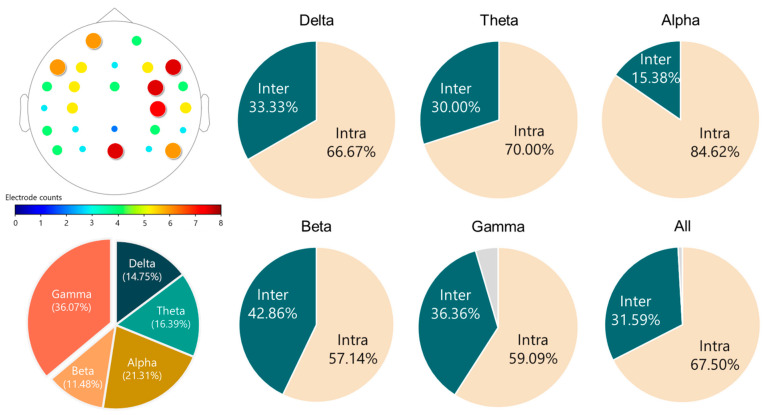
Characteristics of the 61 optimal coherence features selected by the SBS and SVM classifier. Upper-left: The occurring times of each electrode in the 61 features. Bottom-left: Proportion of each band in the 61 features. Remaining: Proportions of the intra-hemispheric (Intra) and inter-hemispheric (Inter) coherence features in each band. The gray part represents the regional connectivity of the electrode pair of FCz and Pz.

Previous literature has shown that MDD affects the functional connectivity of the brain in the resting state, but these findings were still inconclusive. For example, both functional MRI [67] and EEG [68] studies showed that MDD participants had increased connectivity in different brain regions, whereas some EEG studies [69,70] reported that a decreased functional connectivity was found in patients with MDD. This study combined all these coherence features as a feature vector for the MDD-HC classification, and therefore we did not further test whether each coherence had a significant difference between the two groups (MDD versus HC). Recently, Peng et al. [17] classified MDD and HC using the EEG connectivity features from four different bands (delta, theta, alpha, and beta). They concluded that the connectivity features of the four frequency bands are all crucial to the high-performance classification. Their results also suggested that depression affects the connectivity across the whole brain, as nearly all electrode pairs were involved in the connectivity-based classification. Our findings are in line with theirs, as evidenced by the 61 optimal coherence features summarized in Figure 8. There are three main findings. First, all 26 electrodes were used in the 61 optimal features. Second, all of the frequency bands contributed to the optimal coherence feature set. Third, the intra-hemispheric coherence features make up the majority of the optimal coherence feature set.

*Feature Selection*. In the current study, we applied the wrapper approach, i.e., the SBS, to identify the optimal feature subset among the 1859 feature candidates. However, there is a limitation of the SBS algorithm. It is unable to re-evaluate the utility of the features that have been discarded. In other words, the optimal feature subset selected by the SBS algorithm may not really be the optimal. Other greedy and randomized feature selection methods, such as the genetic algorithm (GA), can be used to overcome this limitation. It should also be noted that the optimal feature subset determined by SBS may differ from those identified by other methods like GA.

As previously noted, the aim of the current study is to identify the best features among the 1859 feature candidates. The obtained 62 optimal features are explainable, which have already been able to provide a useful reference for researchers looking to investigate the relationship between them and the pathological mechanism of the brain in the future. If a dimension reduction method is further applied to the 62 features to obtain a lower-dimensional feature representation, not only can the data be visualized, but also the classification accuracy can be improved. A variety of advanced methods, including principal component analysis (PCA) and its nonlinear version, i.e., the kernel PCA, manifold learning approaches: the locally linear embedding (LLE), *t*-distributed stochastic neighbor embedding (t-SNE), the uniform manifold approximation and projection (UMAP), and neural network approaches such as autoencoders and self-organizing map (SOM). Although such research is beyond the scope of this study, it is definitely a worthwhile topic for future research.

## 6. Conclusions

The main goal of the current study was to provide a systematic validation of the efficacy of common and standard EEG features used in previous ML-based MDD-HC classification studies on a relatively large and diverse dataset. To this end, we thoroughly and systematically analyzed these features at the electrode, region, and optimal feature subset levels. Our results revealed that the coherence feature is the most reliable, effective and generalizable solution for the EEG-based MDD detection in real-world applications, where there might be high data variability caused by uncontrollable or unavoidable conditions. We also showed that the CK-SVM, a variant of SVM, was able to improve the performance generalization of the challenging MDD-HC classification, achieving a high “individual-level” classification accuracy of 84% on a large and independent dataset. According to the promising results obtained on a large dataset across multiple EEG recording sites, we have demonstrated the effectiveness and applicability of the EEG- and ML-based computer-aided diagnosis (CADx) for MDD. 

Several critical issues remain to be solved. One issue is the establishment of an age-optimized MDD-HC classification model. Since MDD and mild cognitive impairment (MCI) or early dementia share some behavior signs in their early stage (i.e., cognitive declination), diagnosis of geriatric MDD has been a challenging task in clinical practice. Therefore, to develop an age-optimized CADx that is sensitive to MDD symptoms and disregards potential signal contamination from aging-related changes will be the next challenge. In summary, our results provide a reliable and valuable reference for future research to develop a more accurate and efficient CADx system.

## Figures and Tables

**Table 1:** Demographic data and results of questionnaires.

Variable	MDD, *n* = 200	HC, *n* = 200	*p-*Values	Effect Size
Gender	142 F, 58 M	142 F, 58 M	1.000	0
Age	53.44 (±16.44)	51.24 (±17.85)	0.1969	0.1293
BDI-II	25.79 (±14.30)	4.18 (±6.74)	5.60 × 10^−59^	1.9282
PHQ-9	12.64 (±7.30)	2.09 (±3.73)	4.51 × 10^−54^	1.8150
HADS-A	10.46 (±4.91)	3.53 (±3.37)	1.18 × 10^−46^	1.6425
HADS-D	10.29 (±5.19)	2.50 (±2.85)	7.91 × 10^−56^	1.8556

Note: BDI-II, Becks Depression Inventory–II; PHQ-9, Patient Health Questionnaire; HADS, Hospital Anxiety and Depression Scale.

**Table 2:** Demographic data and scores of questionnaires for participants in the training set.

Variable	MDD (*n* = 140)	HC (*n* = 140)	*p-*Values	Effect Size
Gender	100 F, 40 M	97 F, 43 M	0.7935	0.0474
Age	53.06 (±16.31)	50.83 (±17.64)	0.2734	0.1312
BDI-II	25.82 (±14.23)	3.69 (±5.98)	1.43 × 10^−44^	2.0201
PHQ-9	12.85 (±7.31)	2.00 (±3.52)	1.77 × 10^−40^	1.8855
HADS-A	10.52 (±4.97)	3.19 (±3.05)	4.85 × 10^−37^	1.7720
HADS-D	10.29 (±5.24)	2.32 (±2.71)	4.63 × 10^−41^	1.9046

**Table 3:** Demographic data and scores of questionnaires for participants in the test set.

Variable	MDD (*n* = 60)	HC (*n* = 60)	*p*-Values	Effect Size
Gender	42 F, 18 M	45 F, 15 M	0.6826	0.1091
Age	54.32 (±16.70)	52.18 (±17.40)	0.4983	0.1240
BDI-II	25.70 (±14.46)	5.30 (±8.41)	4.22 × 10^−16^	1.7237
PHQ-9	12.13 (±7.25)	2.30 (±4.18)	4.05 × 10^−15^	1.6475
HADS-A	10.32 (±4.76)	4.32 (±3.91)	1.42 × 10^−11^	1.3662
HADS-D	10.27 (±5.08)	2.90 (±3.12)	3.16 × 10^−16^	1.7334

**Table 4:** Comparison of 5-fold cross validation accuracies among different features, frequency bands, and scalp regions using LDA (in %). In each grid of this table, the numbers inside and outside the brackets denote the accuracies obtained from the features with and without the SBS-based feature selection.

EEG Features		Frontal	Central	Temporal	Parietal	ALL
BP	*δ*	49.53 ± 6.80 (55.82 ± 3.19)	56.35 ± 4.24 (60.77 ± 4.71)	53.86 ± 3.50 (61.19 ± 5.49)	51.80 ± 3.81 (58.09 ± 5.30)	48.80 ± 6.76 (63.07 ± 3.11)
	*θ*	49.63 ± 11.93 (58.91 ± 4.74)	51.24 ± 6.88 (55.26 ± 2.08)	52.62 ± 7.25 (57.25 ± 5.37)	45.83 ± 4.57 (55.44 ± 4.97)	52.58 ± 8.61 (59.82 ± 9.68)
	*α*	47.06 ± 7.21 (56.28 ± 3.85)	51.67 ± 7.20 (54.01 ± 1.82)	44.49 ± 7.36 (49.16 ± 2.90)	46.16 ± 6.84 (54.93 ± 3.19)	42.09 ± 9.95 (57.65 ± 4.32)
	*β*	52.53 ± 5.36 (58.61 ± 5.71)	55.00 ± 7.35 (55.42 ± 3.64)	53.87 ± 6.51 (56.29 ± 3.11)	50.76 ± 7.64 (53.96 ± 2.62)	51.74 ± 9.00 (64.21 ± 4.43)
	*γ*	48.28 ± 7.21 (54.63 ± 4.43)	55.51 ± 2.19 (56.88 ± 4.13)	46.62 ± 8.46 (51.61 ± 3.64)	43.94 ± 6.00 (53.13 ± 5.46)	44.15 ± 11.66 (60.29 ± 3.81)
COH	*δ*	53.44 ± 8.04 (61.94 ± 2.53)	54.38 ± 5.86 (59.88 ± 9.68)	46.70 ± 5.95 (59.08 ± 4.32)	52.85 ± 3.23 (62.83 ± 3.57)	52.49 ± 4.85 (81.27 ± 3.64)
	*θ*	56.30 ± 7.58 (64.24 ± 6.83)	58.64 ± 7.65 (61.49 ± 5.30)	51.86 ± 5.64 (61.53 ± 5.60)	52.47 ± 10.46 (63.58 ± 4.43)	49.16 ± 9.78 (75.36 ± 3.46)
	*α*	52.67 ± 8.48 (66.11 ± 6.43)	56.08 ± 4.45 (62.44 ± 3.11)	56.36 ± 8.28 (65.28 ± 2.62)	52.34 ± 13.05 (63.25 ± 6.64)	51.32 ± 6.17 (79.06 ± 6.25)
	*β*	55.43 ± 6.91 (66.28 ± 4.60)	58.68 ± 2.92 (62.97 ± 5.30)	55.78 ± 7.18 (64.49 ± 1.96)	53.15 ± 6.28 (64.17 ± 6.53)	53.37 ± 7.41 (85.65 ± 10.68)
	*γ*	50.05 ± 6.86 (62.17 ± 6.74)	58.60 ± 8.13 (63.82 ± 4.29)	46.13 ± 5.57 (63.26 ± 8.07)	55.66 ± 5.33 (59.42 ± 4.43)	49.70 ± 5.41 (78.11 ± 4.71)
HFD	*k*max = 50	51.28 ± 6.06 (59.01 ± 8.10)	57.38 ± 3.30 (59.47 ± 4.07)	52.23 ± 7.21 (60.09 ± 5.82)	48.52 ± 6.25 (55.50 ± 4.97)	50.25 ± 6.29 (62.08 ± 5.91)
	*k*max = 100	52.93 ± 6.93 (59.46 ± 6.02)	59.42 ± 3.90 (60.10 ± 2.42)	55.31 ± 5.98 (60.17 ± 4.92)	51.47 ± 3.54 (57.35 ± 4.92)	54.33 ± 4.38 (63.81 ± 6.39)
	*k*max = 150	52.93 ± 6.93 (59.24 ± 4.60)	58.93 ± 3.86 (60.66 ± 3.75)	54.14 ± 8.45 (60.21 ± 3.64)	50.56 ± 5.60 (58.04 ± 5.71)	54.04 ± 4.73 (64.96 ± 6.04)
KFD		44.60 ± 8.37 (58.36 ± 4.60)	41.05 ± 10.18 (63.01 ± 2.90)	35.65 ± 9.99 (58.23 ± 5.58)	50.75 ± 10.15 (54.41 ± 6.87)	46.38 ± 8.46 (61.89 ± 4.87)

**Table 5:** The numbers and the percentages of BP, COH, HFD, and KFD in the optimal feature subset for each classifier.

	*K*-NN	LDA	SVM
BP	3.03% (1)	4.76% (3)	1.61% (1)
COH	96.97% (32)	95.24% (60)	98.39% (61)
HFD	0 (0)	0 (0)	0 (0)
KFD	0 (0)	0 (0)	0 (0)

**Table 6:** Comparison of training and testing classification accuracy among different classifiers (in %).

Classifier (Number of Features)	Training Set	Test Set		
	5-Fold CV Accuracy	(Accuracy	Sensitivity	Specificity)
K-NN ($N_{f}=33$)	66.43 ± 7.79	48.33	48.33	48.33
LDA ($N_{f}=63$)	88.21 ± 5.60	69.17	75.00	63.33
SVM ($N_{f}=51$)	86.07 ± 4.71	80.83	86.67	75.00
SVM ($N_{f}=62$)	87.50 ± 4.92	77.50	85.00	70.00
CK-SVM ($N_{f}=51$)	91.07 ± 3.43	84.16	88.33	80.00
CK-SVM ($N_{f}=62$)	89.28 ± 3.29	80.83	88.33	73.33

**Table 7:** Five-fold cross validation accuracies among different frequency bands and scalp regions using a normalized BP and a LDA classifier (in %). The numbers inside and outside the brackets denote the accuracies obtained from the features with and without the SBS-based feature selection.

EEG Features		Frontal	Central	Temporal	Parietal	ALL
Normalized BP	*δ*	52.14 (56.43)	57.14 (58.57)	53.35 (58.21)	42.86 (51.43)	50.00 (63.57)
	*θ*	53.93 (58.21)	52.86 (54.64)	57.14 (58.21)	43.93 (54.64)	54.29 (60.36)
	*α*	51.43 (58.57)	51.43 (52.86)	46.43 (53.93)	46.43 (53.93)	49.29 (57.50)
	*β*	54.29 (56.07)	56.43 (57.86)	56.07 (57.86)	51.43 (55.00)	52.50 (62.86)
	*γ*	54.29 (58.21)	59.29 (59.29)	50.00 (56.43)	46.79 (55.00)	48.21 (63.21)

## Appendix A

Here, we provide the feature performance ranking results of the features in the optimal feature subsets for the *K*-NN (33 optimal features), LDA (63 optimal features), and SVM (62 optimal features) classifiers in Figure A1, Figure A2 and Figure A3, respectively, where the accuracies are the same as those shown in Figure 5 (Section 4.2). For example, as the total 33 optimal features are used, the 5-fold CV accuracy (66.43) for the K-NN classifier is exactly the same as the one shown in Figure 5. It should also be noted that the feature performance ranking does not refer to the individual feature ranking, because the SBS algorithm is a wrapper feature selection approach, which does not rank each feature but evaluates the classification performance of a group of features. For example, in the case of the *K*-NN classifier, the accuracy associated with the second feature Coh (CP3-FC3, gamma) is the accuracy obtained by using the top two features together, i.e., Coh (F4-FC4, gamma) and Coh (CP3-FC3, gamma).
